# Supplementary material for: Effects of simulated reduced gravity and walking speed on ankle, knee, and hip quasi-stiffness in overground walking
Source: PLoS One. 2022 Aug 9;17(8):e0271927. doi: 10.1371/journal.pone.0271927 (PMC9362947; doi:10.1371/journal.pone.0271927)
Supplement: S1 Table — (DOCX) [file pone.0271927.s001.docx]

**S1 Table. Participant descriptive data.**

| Subject | Height (m) | Leg Length (m) | Weight (N) | Age (years) | Sex |
| --- | --- | --- | --- | --- | --- |
| 1 | 1.8 | 1.02 | 715 | 23 | m |
| 2 | 1.75 | 0.9 | 610 | 24 | f |
| 3 | 1.87 | 1.03 | 594 | 32 | m |
| 4 | 1.67 | 0.82 | 887 | 36 | m |
| 5 | 1.69 | 0.9 | 665 | 24 | f |
| 6 | 1.7 | 0.86 | 617 | 27 | m |
| 7 | 1.78 | 0.95 | 690 | 25 | m |
| 8 | 1.69 | 0.96 | 674 | 25 | f |
| 9 | 1.71 | 0.95 | 638 | 22 | f |
| 10 | 1.65 | 0.87 | 660 | 24 | f |
| 11 | 1.7 | 0.94 | 660 | 31 | f |
| 12 | 1.86 | 1 | 819 | 24 | m |
