# Supplementary material for: Effects of simulated reduced gravity and walking speed on ankle, knee, and hip quasi-stiffness in overground walking
Source: PLoS One. 2022 Aug 9;17(8):e0271927. doi: 10.1371/journal.pone.0271927 (PMC9362947; doi:10.1371/journal.pone.0271927)
Supplement: S2 Table — Numbers in bold indicate conditions where data were lost. (DOCX) [file pone.0271927.s002.docx]

**S2 Table. Walking conditions and number of subjects for which phases of quasi-stiffness could not be calculated.**

| Gravity | Speed (m/s) | Total subjects | Ankle | | | | Knee | | Hip | | | |
| --- | --- | --- | --- | --- | --- | --- | --- | --- | --- | --- | --- | --- |
|  |  |  | K_AnD1_ | K_AnD2_ | K_AnDF_ | K_AnPF_ | K_HiF_ | K_HiE_ | K_HiE1_ | K_HiE2_ | K_HiE_ | K_HiF_ |
| 1 G | 0.4 | 12 | 0 | 0 | 0 | 0 | 0 | 0 | **1** | 0 | 0 | 0 |
|  | 0.8 | 12 | 0 | 0 | 0 | 0 | 0 | 0 | 0 | 0 | 0 | 0 |
|  | 1.2 | **11** | 0 | 0 | 0 | 0 | 0 | 0 | 0 | 0 | 0 | 0 |
|  | 1.6 | 12 | 0 | 0 | 0 | 0 | 0 | 0 | 0 | 0 | 0 | 0 |
| 0.76 G | 0.4 | 12 | 0 | 0 | 0 | 0 | 0 | 0 | 0 | 0 | 0 | 0 |
|  | 0.8 | 12 | 0 | 0 | 0 | 0 | 0 | 0 | 0 | 0 | 0 | 0 |
|  | 1.2 | 12 | 0 | 0 | 0 | 0 | 0 | 0 | 0 | 0 | 0 | 0 |
|  | 1.6 | 12 | 0 | 0 | 0 | 0 | 0 | 0 | 0 | 0 | 0 | 0 |
| 0.54 G | 0.4 | 12 | **1** | **1** | 0 | 0 | 0 | 0 | **1** | **1** | 0 | **1** |
|  | 0.8 | 12 | **1** | **1** | 0 | 0 | 0 | 0 | 0 | 0 | 0 | 0 |
|  | 1.2 | 12 | 0 | 0 | 0 | 0 | 0 | 0 | 0 | 0 | 0 | **1** |
|  | 1.6 | 12 | 0 | 0 | 0 | 0 | 0 | 0 | 0 | 0 | 0 | 0 |
| 0.31 G | 0.4 | 12 | 0 | **2** | 0 | 0 | 0 | 0 | **1** | **2** | 0 | **2** |
|  | 0.8 | 12 | 0 | **4** | 0 | 0 | 0 | 0 | 0 | 0 | 0 | **3** |
|  | 1.2 | 12 | **1** | **1** | 0 | 0 | 0 | 0 | 0 | 0 | 0 | **4** |
|  | 1.6 | **10** | 0 | 0 | 0 | 0 | 0 | 0 | 0 | 0 | 0 | **3** |

Numbers in bold indicate number conditions where quasi-stiffness could not be found for some participants.
