# Supplementary material for: Effects of simulated reduced gravity and walking speed on ankle, knee, and hip quasi-stiffness in overground walking
Source: PLoS One. 2022 Aug 9;17(8):e0271927. doi: 10.1371/journal.pone.0271927 (PMC9362947; doi:10.1371/journal.pone.0271927)
Supplement: S3 Table — (DOCX) [file pone.0271927.s003.docx]

**S3 Table. Mean quasi-stiffness values with standard deviations for all conditions.**

| Gravity | Speed (m/s) | Ankle | | | | Knee | | Hip | | | |
| --- | --- | --- | --- | --- | --- | --- | --- | --- | --- | --- | --- |
|  |  | K_AnD1_ | K_AnD2_ | K_AnDF_ | K_AnPF_ | K_KnF_ | K_KnE_ | K_HiE1_ | K_HiE2_ | K_HiE_ | K_HiF_ |
| 1 G | 0.4 | 0.041 | 0.069 | 0.077 | 0.055 | 0.056 | -0.012 | 0.025 | 0.030 | 0.022 | 0.029 |
|  |  | ±0.075 | ±0.02 | ±0.039 | ±0.018 | ±0.031 | ±0.071 | ±0.012 | ±0.02 | ±0.006 | ±0.026 |
|  | 0.8 | 0.063 | 0.075 | 0.072 | 0.081 | 0.055 | 0.035 | 0.034 | 0.028 | 0.026 | 0.048 |
|  |  | ±0.016 | ±0.009 | ±0.009 | ±0.016 | ±0.031 | ±0.069 | ±0.014 | ±0.009 | ±0.006 | ±0.014 |
|  | 1.2 | 0.056 | 0.108 | 0.076 | 0.068 | 0.060 | 0.053 | 0.061 | 0.030 | 0.030 | 0.076 |
|  |  | ±0.014 | ±0.032 | ±0.012 | ±0.014 | ±0.023 | ±0.032 | ±0.026 | ±0.007 | ±0.006 | ±0.03 |
|  | 1.6 | 0.062 | 0.172 | 0.092 | 0.064 | 0.081 | 0.077 | 0.086 | 0.036 | 0.033 | 0.106 |
|  |  | ±0.011 | ±0.056 | ±0.015 | ±0.008 | ±0.022 | ±0.046 | ±0.045 | ±0.013 | ±0.005 | ±0.028 |
| 0.76 G | 0.4 | 0.038 | 0.060 | 0.062 | 0.050 | 0.042 | -0.020 | 0.014 | 0.041 | 0.018 | 0.033 |
|  |  | ±0.069 | ±0.029 | ±0.044 | ±0.021 | ±0.025 | ±0.05 | ±0.004 | ±0.023 | ±0.005 | ±0.023 |
|  | 0.8 | 0.062 | 0.054 | 0.057 | 0.063 | 0.041 | 0.048 | 0.023 | 0.038 | 0.026 | 0.038 |
|  |  | ±0.013 | ±0.015 | ±0.008 | ±0.014 | ±0.015 | ±0.035 | ±0.006 | ±0.009 | ±0.005 | ±0.014 |
|  | 1.2 | 0.051 | 0.101 | 0.063 | 0.055 | 0.053 | 0.061 | 0.041 | 0.036 | 0.027 | 0.056 |
|  |  | ±0.013 | ±0.054 | ±0.012 | ±0.011 | ±0.014 | ±0.024 | ±0.018 | ±0.011 | ±0.004 | ±0.028 |
|  | 1.6 | 0.052 | 0.122 | 0.070 | 0.051 | 0.063 | 0.065 | 0.059 | 0.035 | 0.028 | 0.108 |
|  |  | ±0.008 | ±0.119 | ±0.014 | ±0.01 | ±0.014 | ±0.024 | ±0.022 | ±0.012 | ±0.005 | ±0.032 |
| 0.54 G | 0.4 | 0.035 | 0.045 | 0.060 | 0.033 | 0.038 | -0.040 | 0.026 | 0.059 | 0.027 | 0.031 |
|  |  | ±0.06 | ±0.025 | ±0.044 | ±0.021 | ±0.018 | ±0.041 | ±0.032 | ±0.061 | ±0.021 | ±0.014 |
|  | 0.8 | 0.052 | 0.032 | 0.049 | 0.041 | 0.037 | 0.036 | 0.019 | 0.041 | 0.023 | 0.031 |
|  |  | ±0.016 | ±0.016 | ±0.019 | ±0.014 | ±0.01 | ±0.06 | ±0.006 | ±0.02 | ±0.004 | ±0.013 |
|  | 1.2 | 0.053 | 0.040 | 0.050 | 0.037 | 0.042 | 0.033 | 0.027 | 0.039 | 0.025 | 0.051 |
|  |  | ±0.014 | ±0.057 | ±0.019 | ±0.014 | ±0.009 | ±0.048 | ±0.009 | ±0.018 | ±0.004 | ±0.025 |
|  | 1.6 | 0.047 | -0.007 | 0.039 | 0.030 | 0.050 | 0.035 | 0.038 | 0.038 | 0.027 | 0.095 |
|  |  | ±0.012 | ±0.067 | ±0.039 | ±0.008 | ±0.011 | ±0.067 | ±0.014 | ±0.019 | ±0.006 | ±0.026 |
| 0.31 G | 0.4 | 0.036 | 0.038 | 0.033 | 0.013 | 0.022 | -0.030 | 0.029 | 0.038 | 0.036 | 0.024 |
|  |  | ±0.048 | ±0.031 | ±0.049 | ±0.018 | ±0.009 | ±0.034 | ±0.012 | ±0.045 | ±0.018 | ±0.029 |
|  | 0.8 | 0.047 | 0.013 | 0.043 | 0.017 | 0.026 | 0.023 | 0.017 | 0.035 | 0.025 | 0.022 |
|  |  | ±0.013 | ±0.009 | ±0.015 | ±0.007 | ±0.01 | ±0.049 | ±0.006 | ±0.03 | ±0.005 | ±0.012 |
|  | 1.2 | 0.049 | 0.003 | 0.042 | 0.018 | 0.035 | 0.029 | 0.022 | 0.031 | 0.026 | 0.040 |
|  |  | ±0.02 | ±0.032 | ±0.021 | ±0.01 | ±0.01 | ±0.051 | ±0.005 | ±0.03 | ±0.005 | ±0.025 |
|  | 1.6 | 0.043 | -0.023 | 0.035 | 0.015 | 0.034 | 0.017 | 0.030 | 0.047 | 0.028 | 0.068 |
|  |  | ±0.017 | ±0.033 | ±0.025 | ±0.004 | ±0.012 | ±0.046 | ±0.011 | ±0.025 | ±0.006 | ±0.04 |
