# Supplementary material for: Effects of simulated reduced gravity and walking speed on ankle, knee, and hip quasi-stiffness in overground walking
Source: PLoS One. 2022 Aug 9;17(8):e0271927. doi: 10.1371/journal.pone.0271927 (PMC9362947; doi:10.1371/journal.pone.0271927)
Supplement: S4 Table — (DOCX) [file pone.0271927.s004.docx]

**S4 Table. Mean R^2^ for each quasi-stiffness linear model with standard deviations for all conditions.**

| Gravity | Speed (m/s) | Ankle | | | | Knee | | Hip | | | |
| --- | --- | --- | --- | --- | --- | --- | --- | --- | --- | --- | --- |
|  |  | K_AnD1_ | K_AnD2_ | K_AnDF_ | K_AnPF_ | K_KnF_ | K_KnE_ | K_HiE1_ | K_HiE2_ | K_HiE_ | K_HiF_ |
| 1 G | 0.4 | 0.804 | 0.951 | 0.793 | 0.853 | 0.855 | 0.490 | 0.833 | 0.859 | 0.871 | 0.675 |
|  |  | ±0.166 | ±0.042 | ±0.281 | ±0.051 | ±0.184 | ±0.207 | ±0.232 | ±0.174 | ±0.154 | ±0.244 |
|  | 0.8 | 0.969 | 0.986 | 0.978 | 0.907 | 0.856 | 0.660 | 0.955 | 0.940 | 0.962 | 0.834 |
|  |  | ±0.026 | ±0.013 | ±0.017 | ±0.041 | ±0.272 | ±0.251 | ±0.04 | ±0.044 | ±0.017 | ±0.157 |
|  | 1.2 | 0.973 | 0.958 | 0.956 | 0.957 | 0.931 | 0.877 | 0.949 | 0.892 | 0.934 | 0.894 |
|  |  | ±0.03 | ±0.058 | ±0.025 | ±0.021 | ±0.144 | ±0.191 | ±0.033 | ±0.049 | ±0.029 | ±0.068 |
|  | 1.6 | 0.982 | 0.897 | 0.887 | 0.971 | 0.979 | 0.933 | 0.926 | 0.846 | 0.898 | 0.920 |
|  |  | ±0.031 | ±0.104 | ±0.086 | ±0.021 | ±0.012 | ±0.037 | ±0.049 | ±0.104 | ±0.05 | ±0.103 |
| 0.76 G | 0.4 | 0.755 | 0.960 | 0.768 | 0.798 | 0.901 | 0.638 | 0.797 | 0.789 | 0.814 | 0.807 |
|  |  | ±0.23 | ±0.032 | ±0.278 | ±0.088 | ±0.094 | ±0.2 | ±0.146 | ±0.24 | ±0.153 | ±0.18 |
|  | 0.8 | 0.955 | 0.981 | 0.976 | 0.922 | 0.926 | 0.775 | 0.941 | 0.965 | 0.954 | 0.908 |
|  |  | ±0.055 | ±0.02 | ±0.012 | ±0.04 | ±0.131 | ±0.224 | ±0.051 | ±0.03 | ±0.025 | ±0.063 |
|  | 1.2 | 0.982 | 0.906 | 0.945 | 0.959 | 0.982 | 0.894 | 0.911 | 0.914 | 0.934 | 0.933 |
|  |  | ±0.014 | ±0.128 | ±0.032 | ±0.026 | ±0.01 | ±0.094 | ±0.064 | ±0.073 | ±0.031 | ±0.075 |
|  | 1.6 | 0.969 | 0.716 | 0.789 | 0.980 | 0.978 | 0.876 | 0.883 | 0.849 | 0.894 | 0.947 |
|  |  | ±0.037 | ±0.231 | ±0.168 | ±0.013 | ±0.015 | ±0.081 | ±0.061 | ±0.084 | ±0.036 | ±0.055 |
| 0.54 G | 0.4 | 0.746 | 0.886 | 0.701 | 0.793 | 0.900 | 0.730 | 0.660 | 0.863 | 0.747 | 0.776 |
|  |  | ±0.214 | ±0.087 | ±0.321 | ±0.192 | ±0.084 | ±0.162 | ±0.185 | ±0.108 | ±0.189 | ±0.187 |
|  | 0.8 | 0.938 | 0.849 | 0.913 | 0.930 | 0.965 | 0.757 | 0.915 | 0.934 | 0.920 | 0.926 |
|  |  | ±0.063 | ±0.201 | ±0.073 | ±0.072 | ±0.036 | ±0.19 | ±0.049 | ±0.115 | ±0.037 | ±0.072 |
|  | 1.2 | 0.959 | 0.679 | 0.841 | 0.975 | 0.969 | 0.833 | 0.927 | 0.958 | 0.950 | 0.969 |
|  |  | ±0.033 | ±0.295 | ±0.241 | ±0.027 | ±0.015 | ±0.126 | ±0.037 | ±0.031 | ±0.024 | ±0.03 |
|  | 1.6 | 0.963 | 0.510 | 0.660 | 0.988 | 0.973 | 0.739 | 0.908 | 0.911 | 0.927 | 0.893 |
|  |  | ±0.028 | ±0.282 | ±0.24 | ±0.008 | ±0.012 | ±0.257 | ±0.054 | ±0.07 | ±0.044 | ±0.098 |
| 0.31 G | 0.4 | 0.743 | 0.923 | 0.786 | 0.844 | 0.863 | 0.688 | 0.751 | 0.699 | 0.755 | 0.756 |
|  |  | ±0.239 | ±0.045 | ±0.208 | ±0.165 | ±0.111 | ±0.216 | ±0.237 | ±0.218 | ±0.201 | ±0.165 |
|  | 0.8 | 0.880 | 0.696 | 0.863 | 0.873 | 0.919 | 0.676 | 0.856 | 0.753 | 0.847 | 0.894 |
|  |  | ±0.097 | ±0.316 | ±0.091 | ±0.167 | ±0.113 | ±0.171 | ±0.112 | ±0.228 | ±0.087 | ±0.095 |
|  | 1.2 | 0.866 | 0.665 | 0.669 | 0.951 | 0.963 | 0.654 | 0.906 | 0.756 | 0.906 | 0.855 |
|  |  | ±0.126 | ±0.226 | ±0.253 | ±0.088 | ±0.024 | ±0.25 | ±0.05 | ±0.253 | ±0.055 | ±0.176 |
|  | 1.6 | 0.957 | 0.698 | 0.521 | 0.977 | 0.954 | 0.686 | 0.907 | 0.913 | 0.938 | 0.829 |
|  |  | ±0.039 | ±0.145 | ±0.263 | ±0.021 | ±0.033 | ±0.237 | ±0.067 | ±0.097 | ±0.037 | ±0.19 |
